# ﻿A new typical cavefish of the genus *Triplophysa* (Teleostei, Cypriniformes, Nemacheilidae) from the Jinsha River, Yunnan, China

**DOI:** 10.3897/zookeys.1253.153155

**Published:** 2025-09-23

**Authors:** Min Shi, Yuan-Chao Chen, Xing-Jin Che, Wei Dao, Wen-Ming Liu, Deng-Shan Wang, Jun-Xing Yang, Xiao-Ai Wang

**Affiliations:** 1 Yunnan Key Laboratory of Plateau Fish Breeding/Yunnan Engineering Research Center for Plateau-Lake Health and Restoration, Kunming lnstitute of Zoology, Chinese Academy of Sciences, Kunming, Yunnan, China Kunming lnstitute of Zoology, Chinese Academy of Sciences Kunming China; 2 University of Chinese Academy of Sciences, Beijing, China University of Chinese Academy of Sciences Beijing China; 3 Fisheries Management Station, Zhaotong, Yunnan, China Fisheries Management Station Zhaotong China

**Keywords:** Cavefish, Cyt *b* sequence, loach, molecular phylogeny, new species, *
Triplophysa
*

## Abstract

*Triplophysa baishuijiangensis***sp. nov.**, a new cave-dwelling loach, was collected from an underground river outlet in Xiaoganxi Village, Yiliang County, Yunnan Province, China, situated within the core area of the Baishuijiang National Aquatic Germplasm Resources Reserve for Endemic Fish, part of the Hengjiang–Jinsha River system. The new species is distinguished from congeners by significant genetic divergence and the following combination of characteristics: vestigial eyes, absence of skin pigmentation, pelvic fin tip reaching the anus, complete lateral line, developed posterior chamber of the swim bladder, and dorsal fin rays iii-7. Phylogenetic analysis based on Cyt *b* positions this new species in the hypogean group of *Triplophysa*, increasing the documented cave species in the genus from 42 to 43. *Triplophysa baishuijiangensis***sp. nov.** represents the first documented typical cavefish in the Jinsha River basin, underscoring the region’s ecological significance for subterranean adaptation and suggesting unexplored cave biodiversity that merits systematic investigation.

## ﻿Introduction

*Triplophysa* Rendahl, 1933, the most species-rich genus in the family Nemacheilidae, comprises approximately 166 valid species, with 78% (130 species) distributed in China (Fricke et al. 2025). These species are primarily distributed across the Qinghai-Tibetan Plateau and adjacent regions but are also found in southwestern and northern China (Zhu 1989; Chen et al. 2009; Tang et al. 2021). Morphological characteristics distinguishing *Triplophysa* from other genera in the Nemacheilidae include: anterior and posterior nostrils closely set, a posterior wall of the bony swim bladder capsule, body cylindrical anteriorly, progressively compressed posteriorly, and sexual dimorphism (tubercles and thickened pads on the snout, cheeks, and dorsal surfaces of pectoral fins) (Zhu 1989). Within *Triplophysa*, sexual dimorphism presents various manifestations, Hou et al. (2010) categorized these into 12 distinct types, notably, of which one demonstrates sexual dimorphism in head and snout shape: males possess a squarish snout (in dorsal view), while females exhibit a rounded snout.

*Triplophysa* species are classified into hypogean (cave-dwelling) and epigean (surface-dwelling) groups based on ecological habits (Liu et al. 2022; Lu et al. 2022). Recent taxonomic studies have confirmed the monophyly of these two groups, with the epigean group occupying a basal position within *Triplophysa* (Yan 2018; Chen et al. 2019; Liu et al. 2022; Luo et al. 2023a; Lan et al. 2024; Cao et al. 2025). Among the hypogean group, varying degrees of ocular and pigment degeneration are observed, ranging from atypical cavefish to fully troglomorphic forms (Yan 2018; Liu et al. 2022). Typical cavefish exhibit advanced troglomorphic adaptations, characterized by regressed or completely degenerated eyes, translucent bodies, and well-developed sensory barbels (Li 2018).

Another Nemacheilid genus, *Claea* Kottelat, 2011, is morphologically similar to *Triplophysa*, with frequent confusion between the two genera (Kottelat, 2011; Chen et al. 2021; Zhang et al. 2024). Recent phylogenies confirm their close affinity: *Claea* forms a sister clade to the *Triplophysa* hypogean group and is embedded within *Triplophysa* (Yan 2017; Zhang et al. 2024; Lei et al. 2025). *Claea* species show a preference for cave habitats (Liao et al. 1997; Chen et al. 2021), with one troglomorphic species (*C. scet* Lei, He, Huang, Zhou & He, 2025) described. Thus, including *Claea* in phylogenetic analyses of putative new *Triplophysa* cave species is essential to avoid misidentification.

The karst region of southwestern China (Yunnan, Guizhou, Guangxi, and surrounding areas) is endowed with abundant river systems such as such as the Wujiang (a tributary of the Yangtze River), Nanpanjiang, Beipanjiang, and Hongshui River. Its diverse karst landforms (Liu et al. 2008) provide a favorable environment for the evolution of cavefish (Yan 2018; Luo et al. 2023b), and this area also serves as a biodiversity hotspot for cave-dwelling loaches, with 83 species of cave-adapted Nemacheilidae fishes currently documented (Luo et al. 2023c). Among these, the genus *Triplophysa* represents the largest group, comprising 43 species (Lan et al. 2024; Cao et al. 2025; Suppl. material 1).

Currently, six cave-dwelling *Triplophysa* species have been described from the Yangtze River basin, with their distributions restricted to the Wujiang River and Yuanjiang River systems. These include *T. qingzhenensis* Liu, Zeng & Gong, 2022, *T. wudangensis* Liu, Zeng & Gong, 2022, *T. rosa* Chen & Yang, 2005, and *T. qini* Deng, Wang & Zhang, 2022 from the Wujiang River, with *T. xiangxiensis* (Yang, Yuan & Liao, 1986) and *T. erythraea* Liu & Huang, 2019 from the Yuanjiang River.

In 2023, 11 cave loach specimens were collected from a karst cave in Xiaoganxi Village, Yiliang County, Yunnan Province, China. This cave is hydrologically connected to the Xiaoganxi stream, which drains into the Baishuijiang River (Jinsha River basin). Based on these specimens exhibiting: closely set anterior and posterior nostrils, cylindrical anterior body progressively compressed posteriorly, and presence of sexual dimorphism, we assign them to *Triplophysa*. Molecular phylogenetic analyses and morphological comparisons demonstrated that these specimens represent a distinct species within the genus *Triplophysa*. Here, we formally describe it as *Triplophysa baishuijiangensis* sp. nov.

## ﻿Materials and methods

Specimens were collected using dip nets at a subterranean river outlet in the Baishuijiang Reserve. Samples were either fixed in 75% ethanol or transported live to the laboratory. After live photos were taken, live samples were fixed in 10% formalin. Before fixation, the right pelvic fin rays were clipped from all samples for molecular analysis and preserved in anhydrous ethanol. All specimens are preserved at the Kunming Institute of Zoology, Chinese Academy of Sciences, Kunming, Yunnan Province, China (**KIZ**).

Morphological examination. Ten specimens were measured with digital calipers, all measurable parameters were recorded as point-to-point distances to an accuracy of 0.01 mm, following the methodology of Li (2018) and Wang (2022). Sex differentiation is based on head shape, referring to the sexual dimorphism description of *T. daqiaoensis* Ding, 1993 in Hou et al. (2010). The count of vertebrae was taken from X-ray photographs (Digital Cabinet X-ray System Xpeart 80, Kubtec Scientific, Stratford, USA). One non-type specimen was dissected to observe and describe the morphology of the intestine and swim bladder, and to count the number of inner gill rakers on the first gill arch.

Comparative morphological data were acquired from specimen examination and literature (Suppl. material 2), type specimens of 14 cave-dwelling *Triplophysa* species were examined in this study, including: *T. shilinensis* Chen & Yang, 1992, *T. anshuiensis* Wu, Wei, Lan & Du, 2018, *T. gejiuensis* (Chu & Chen, 1979), *T. huapingensis* Zheng, Yang & Chen, 2012, *T. nandanensis* Lan, Yang & Chen, 1995, *T. longliensis* Ren, Yang & Chen, 2012, *T. guizhouensis* Wu, He, Yang & Du, 2018, *T. rosa*, *T. tianlinensis* Li, Li, Lan & Du, 2016, *T. tianeensis* Chen, Cui & Yang, 2004, *T. macrocephala* Yang, Wu & Yang, 2012, *T. luochengensis* Li, Lan, Chen & Du, 2017, *T. longipectoralis* Zheng, Du, Chen & Yang, 2009, and *T. xichouensis* Liu, Pan, Yang & Chen, 2017.

Phylogenetic analyses. Genomic DNA was extracted from pelvic fin tissues of four specimens using the Ezup column animal genome DNA extraction kit according to the manufacturer’s protocol. Primers L14724 (5’-GACTTGAAAAACCACCGTTG-3’) and H15915 (5’-CTCCGATCTCCGGATTACAAGAC-3’) were selected for PCR amplification of the mitochondrial cytochrome *b* (Cyt *b*) (Xiao et al. 2001). The amplification conditions were as follows: initial denaturation at 95 °C for 5 min, followed by 35 cycles of 94 °C for 1 min (denaturation), 48 °C for 1 min (annealing), and 72 °C for 1 min (extension), with a final extension at 72 °C for 10 min. The PCR products were sent to Sangon Biotech for sequencing.

A total of 43 Cyt *b* sequences were included in the molecular analysis, comprising four newly sequenced specimens and 39 sequences downloaded from GenBank (Table 1), with *Homatula tigris* Che, Dao, Chen, Pan, Hua, Liang & Wang, 2023 and *H. laxiclathra* Gu & Zhang, 2012 designated as outgroups. Cyt *b* sequences were aligned using the Clustal W algorithm in MEGA v.11.0 (Tamura et al. 2021), followed by manual trimming of the alignment. Genetic distances were calculated using the Kimura-2-parameter model in MEGA v.11.0. Phylogenetic trees were constructed with two methods: Maximum Likelihood (ML) analysis was conducted in IQ-TREE v. 2.1.4 (Minh et al. 2020) with 10,000 ultrafast bootstrap replicates (Hoang et al. 2018), model selection via ModelFinder identified GTR+F+I+G4 as the optimal evolutionary model based on BIC optimization (Kalyaanamoorthy et al. 2017). Bayesian Inference (BI) was performed using MrBayes v. 3.2.7 (Ronquist et al. 2012) with substitution models optimized through PartitionFinder v. 2.1.1 (Lanfear et al. 2017) under the Bayesian Information Criterion, the Cyt *b* gene was partitioned by codon position, with best-fit models assigned as: first codon: TRNEF+I+G, second codon: HKY+I, third codon: TIM+I+G. Two independent runs were executed for 20 million generations each, sampling trees every 1,000 generations. After discarding the initial 25% of samples as burn-in.

**Table 1.** GenBank accession numbers for species used in the molecular phylogenetic analysis.

Species	Localities	Voucher ID	Accession number	Reference
*T. anlongensis*	Xinglong Town, Anlong County, Guzihou, China	GZNU20230112002	OQ754139.1	Luo et al. 2023
*T. baishuijiangensis 1*	Niujie Town, Yiliang County, Yunnan, China	KIZ2025000165	PQ199313	This study
*T. baishuijiangensis 2*	Niujie Town, Yiliang County, Yunnan, China	KIZ2025000166	PQ199314	This study
*T. baishuijiangensis 3*	Niujie Town, Yiliang County, Yunnan, China	KIZ2025000175	PQ199315	This study
*T. baotianensis*	Baotian Town, Panzhou City, Guzihou, China	GZNU20180421005	NC_056365.1	Wang et al. 2021
*T. cehengensis*	Rongbei Town, Ceheng County, Guzihou, China	GZNU20230109001	OQ754132.1	Luo et al. 2023
*T. erythraea*	Dalong Cave, Huayuan County, Hunan, China	HY18011301	MG967615.1	Unpublished
*T. fengshanensis*	Fengshan county, Guangxi, China	SWU20160518002	OQ998929.1	Zhang et al. 2024
*T. guizhouensis*	Lewang Town, Wangmo County, Guzihou, China	GZNU20220313001	OQ241174.1	Luo et al. 2023
*T. huapingensis*	Huaping Town, Leye County, Guangxi, China	GZNU20230404004	OQ754125.1	Luo et al. 2023
*T. langpingensis*	Longping Township, Tianlin County, Guangxi	GZNU20230404001	OQ754122.1	Luo et al. 2023
*T. longipectoralis*	Huanjiang county, Guangxi, China	SWU20161110005	OQ998928.1	Zhang et al. 2024
*T. longliensis*	Longli county, Guizhou, China	SWU20160903003	MW582825.1	Cheng et al. 2021
*T. macrocephala*	Lihu Town, Nandan County, Guangxi, China	GZNU20230404002	OQ754123.1	Luo et al. 2023
*T. nandanensis*	Liuzhai Town, Nandan County, Guangxi, China	GZNU20230404007	OQ754128.1	Luo et al. 2023
*T. nanpanjiangensis*	China	KIZ20080361	MG238302.1	Min et al. 2023
*T. nasobarbatula*	Dongtang Township, Libo County, Guizhou, China	GZNU20220313011	OQ241176.1	Luo et al. 2023
*T. orientalis*	Tibet, China	H234	MK655279.1	Unpublished
*T. panzhouensis*	Hongguo Town, Panzhou City, Guizhou, China	GZNU20220513001	OQ754119.1	Luo et al. 2023
*T. pappenheimi*	Gansu province, China	CF2481hh	KX373843.1	Unpublished
*T. qingzhenensis*	Qingzhen County, Guiyang City, Guizhou, China	IHB201911150004	MT700458.1	Liu et al. 2023
*T. qini*	Houping Village, Wulong County, Chongqing, China	WNHM10234	ON528185.1	Deng et al. 2022
*T. qiubeiensis*	Nijiao Village, Qiubei County, Yunnan, China	GZNU20230404006	OQ754127.1	Luo et al. 2023
*T. robusta*	Tibet, China	H30	OP616095.1	Unpublished
*T. rongduensis*	Rongdu Town, Ceheng County, Guizhou, China	GZNU20230110001	OQ754135.1	Luo et al. 2023
*T. rosa*	HuoLuTown, Wulong County, Chongqing, China	T21	OQ754130.1	Luo et al. 2023
*T. sanduensis*	Zhonghe Town, Sandu County, Guizhou, China	SWU20170613001	MW582822.1	Cheng et al. 2021
*T. stolickai*	China	H9	OP616074.1	Unpublished
*T. tianeensis*	Bala Township, Tian ‘e County, Guangxi, China	GZNU20230404003	OQ754124.1	Luo et al. 2023
*T. wenshanensis*	Dehou Town,Wenshan County, Yunnan, China	JWS2023003	PP661513	Cao et al. 2025
*T. wudangensis*	Wudang District, Guiyang City, Guizhou, China	IHB2019080904	MT700460.1	Liu et al. 2022
*T. xiangxiensis*	Feihu Cave, Hunan Province	/	JN696407.1	Yao et al. 2012
*T. xuanweiensis*	Tangtang Town, Xuanwei City, Yunnan, China	ASIZB223820	OL675198.1	Lu et al. 2022
*T. yaluwang*	Maoying Town, Ziyun City, Guizhou, China	GZNU20240118005	PQ117067	Lan et al. 2024
*T. yangi*	Wulong Town, Shizong County, Yunnan, China	/	PQ356185.2	Cao et al. 2025
*T. zhenfengensis*	Xinlongchang Town, Xingren City, Guizhou, China	GZNU20220313005	OQ241180.1	Luo et al. 2023
*T. ziyunensis*	Maoying Town, Ziyun City, Guizhou, China	GZNU20230529003	PQ117069	Lan et al. 2024
*Claea wulongensis*	Wulong County, Chongqing, China	SWU2019051308	MW582823.1	Cheng et al. 2021
*C. dabryi 1*	Niujie Town, Yiliang County, Yunnan, China	KIZ20230516001	PQ199316	This study
*C. dabryi 2*	Kanding City, Sichuan, China	CWNU201407121	KX289615.1	Unpublished
*C. dabryi 3*	Weixi County, Yunnan, China	KIZ2009003611	MG238216.1	Min et al. 2023
*Homatula tigris*	Panlong District, Kunming City, Yunnan, China	KIZ202100004147	ON124934.1	Che et al. 2023
*H. laxiclathra*	Jiangkou Town, Ningshan County, Shanxi, China	KIZ2005014383	MG238220.1	Min et al. 2023

## ﻿Results

### ﻿Phylogenetic analyses and genetic divergence

**Figure 1.**
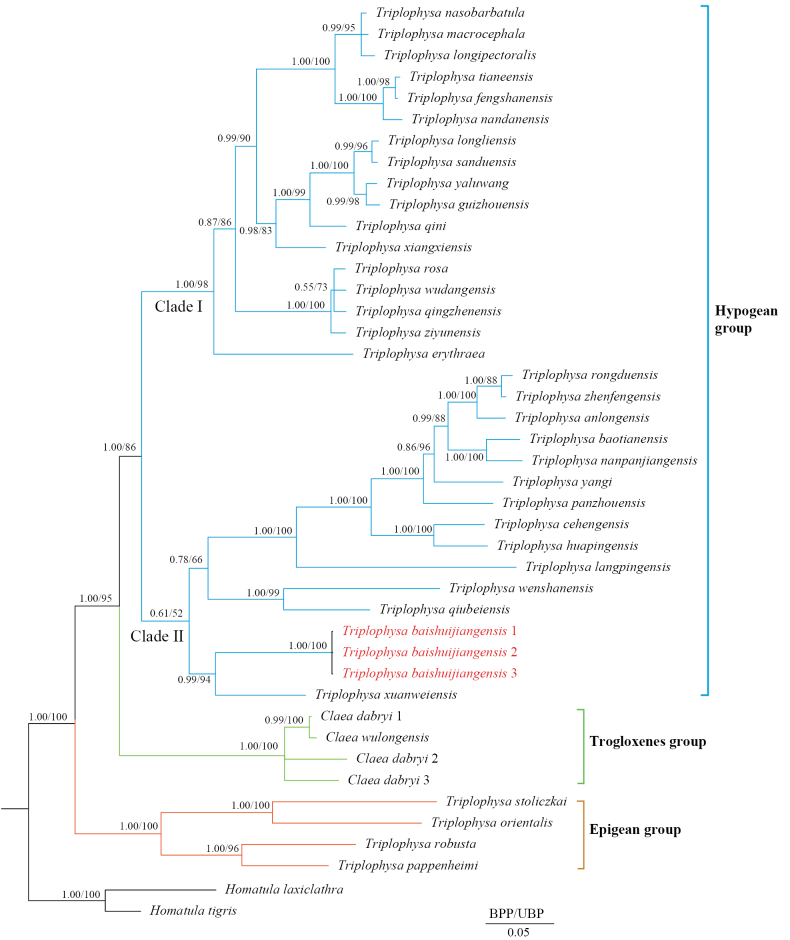
Phylogenetic tree based on 43 Cyt *b* sequences, Bayesian posterior probabilities (BPP) from BI analysis, and ultrafast bootstrap supports (UBP) from ML analysis are shown at nodes.

A total of 43 aligned Cyt *b* gene sequences were used for phylogenetic analysis and genetic distance calculation. The BI and ML phylogenetic trees showed a highly consistent topology, species in phylogenetic tree were classified into three distinct groups: hypogean group, epigean group, and trogloxene group (Fig. 1). The hypogean group comprises 31 cave-dwelling species of *Triplophysa*, while the trogloxene group includes *C. dabryi* (Sauvage, 1874) and *C. wulongensis* (Chen, Sheraliev, Shu & Peng, 2021). Phylogenetic analyses support a sister-group relationship between *Claea* and hypogean *Triplophysa* lineages. The epigean group comprises four surface-dwelling *Triplophysa* species: *T. stolickai* (Steindachner, 1866), *T. orientalis* (Herzenstein, 1888), *T. robusta* (Kessler, 1876), and *T. pappenheimi* (Fang, 1935). The hypogean group is further divided into two clades: Clade II includes 14 species primarily distributed in the upper reaches of the Pearl River (except for *T. baishuijiangensis* sp. nov. in the upper Yangtze River), while Clade I consists of 17 species occurring in the relatively lower reaches of the Yangtze and the Pearl River (Figs 1, 5). Notably, the three samples of *T. baishuijiangensis* sp. nov. form a monophyletic clade with a bootstrap value of 100 and are sister to *T. xuanweiensis* Lu, Li, Mao & Zhao, 2022 within Clade II.

Pairwise genetic distances (Suppl. material 3) between the new species and its congeners within the hypogean group ranged from 10.00% (*T. xuanweiensis*) to 17.42% (*T. anlongensis* Lan, Song, Luo, Zhao, Xiao & Zhou, 2023). Within the hypogean group, the maximum pairwise genetic distance was 19.70% (between *T. fengshanensis* Lan, 2013 and *T. anlongensis*), while the minimum was 0.35% (between *T. tianeensis* and *T. fengshanensis*).

### ﻿Taxonomy

#### ﻿ Triplophysa baishuijiangensis

Taxon classificationAnimaliaCypriniformesNemacheilidae

Shi, Chen, Yang & X.-A. Wang sp. nov.

9CBCB7B0-7F40-5A0C-AAE6-3606589F7F02

https://zoobank.org/8DE249E6-3B59-46D6-BB24-8ECA30E874E5

**Figure 2.**
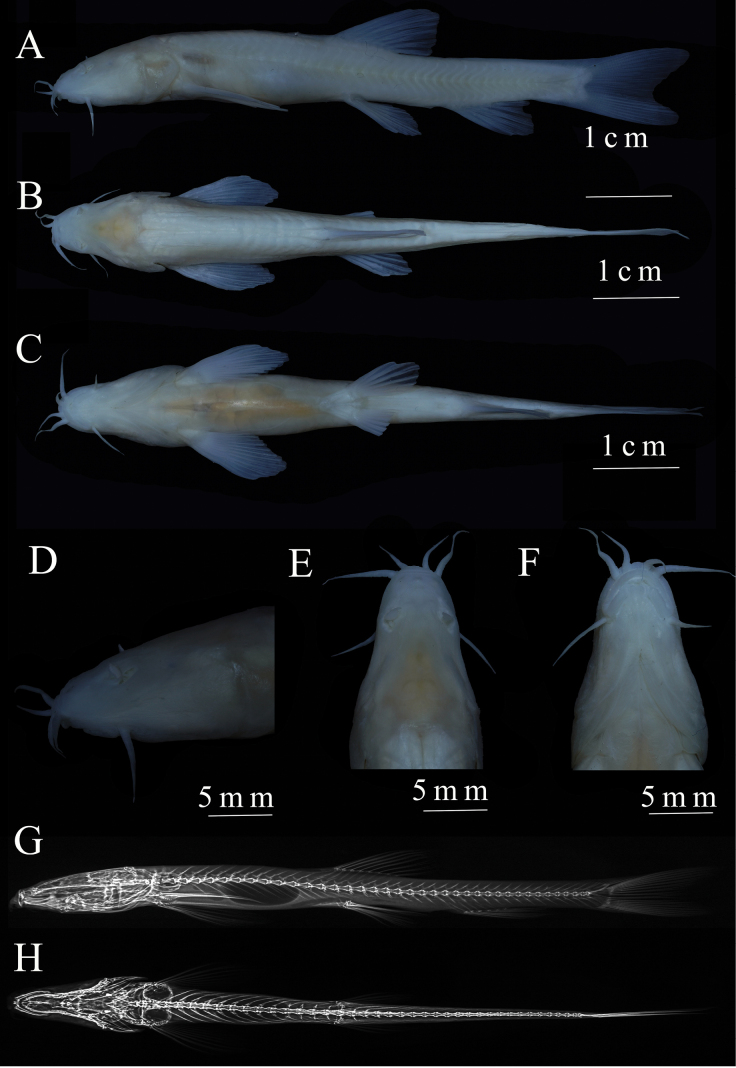
Morphological characteristics of holotype KIZ2025000165 of *Triplophysa baishuijiangensis* sp. nov. A. Lateral view; B. Dorsal view; C. Ventral view; D. Lateral view of head; E. Dorsal view of head; F. Ventral view of head; G. Lateral and; H. Dorsal views of the X-ray graph.

**Figure 3.**
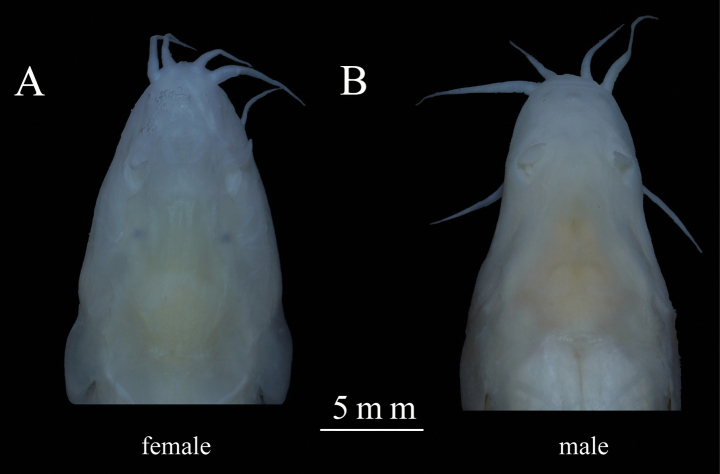
Sexual dimorphism of *Triplophysa baishuijiangensis* sp. nov. A. Female; B. Male.

**Figure 4.**
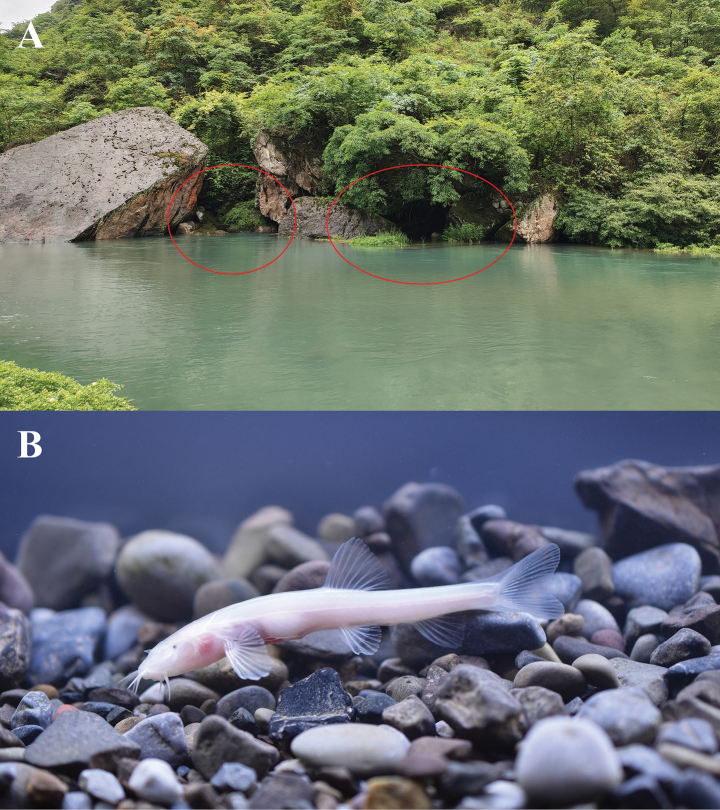
A. Habitat; B. Live photo of *Triplophysa baishuijiangensis* sp. nov. Red circles indicate the subterranean river outlet.

**Figure 5.**
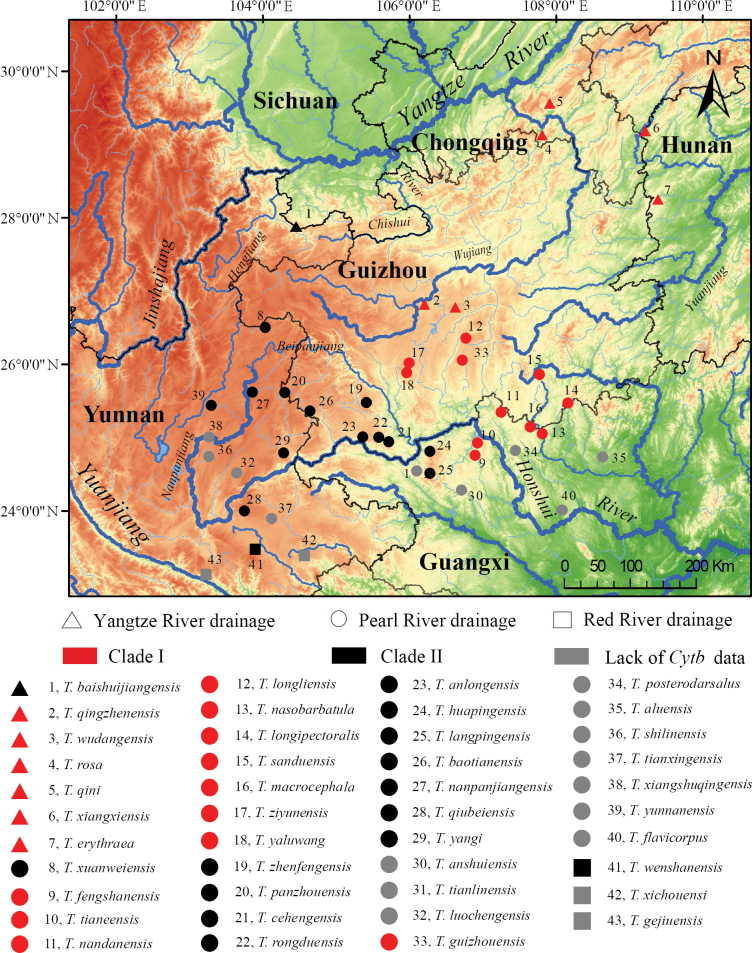
Distribution map of 43 cave-dwelling *Triplophysa* species. Symbols denote species distribution: triangle (Yangtze River), circle (Pearl River), square (Red River). Colors represent phylogenetic clades: red (Clade I), black (Clade II), grey (lack of Cyt b data).

Figs 1, 2, 3, 4, 5, Table 2

##### Type material.

***Holotype*.** • KIZ2025000165 (Fig. 2), 75.64 mm total length, 61.5 mm standard length (SL), collected by Min Shi, Long Zhu, Yuan-Chao Chen, Jian-Fu Wei on May 17, 2023, at Xiaoganxi Village, Niujie town, Yiliang County, Yunnan Province, China, an outlet of a subterranean river belonging to the Baishui River system, Jingsha River drainage (27.82093589°N, 104.47385841°E; ca 628 m. a.s.l.; Fig. 5). ***Paratypes*.** • KIZ2025000175, collected on March 11, 2023; KIZ2025000166–174 (9 specimens) collected on May 17. 31.7–64.7 mm SL, other collection information same as holotype.

##### Diagnosis.

The diagnostic characters for cave-dwelling species of *Triplophysa* are provided in Suppl. material 2. *Triplophysa baishuijiangensis* sp. nov. is distinguished from its congeners by the following combination of characters:

1. Vestigial eyes (vs absent or normal in *T. nanpanjiangensis* Zhu & Cao, 1988, *T. huapingensis*, *T. sanduensis* Chen & Peng, 2019, *T. rongduensis* Mao, Zhao, Yu, Xiao & Zhou, 2023, *T. panzhouensis* Yu, Luo, Lan, Xiao & Zhou, 2023, *T. anlongensis*, *T. baotianensis* Li, Liu & Li, 2018, *T. xiangshuiqingensis* Li, 2004, *T. zhenfengensis* Wang & Li, 2001, *T. nandanensis*, *T. tianxingensis* Yang, Li & Chen, 2016, *T. guizhouensis*, *T. nasobarbatula* Wang & Li, 2001, *T. longliensis*, *T. fengshanensis*, *T. posterodorsalus* (Li, Ran & Chen, 2006), *T. qini*, *T. qiubeiensis* Li & Yang, 2008, *T. shilinensis*, *T. xuanweiensis*, *T. erythraea*, *T. anshuiensis*, *T. xiangxiensis*, *T. gejiuensis*, *T. flavicorpus* Yang, Chen & Lan, 2004, *T. yunnanensis* Yang, 1990, and other epigean congeners);
2. Skin pigment absent (vs reduced or covers the whole body in *T. luochengensis*, *T. qingzhenensis*, *T. macrocephala*, *T. wudangensis*, *T. longipectoralis*, *T. xichouensis*, *T. ziyunensis* Wu, Luo, Xiao & Zhou, 2024, *T. yaluwang* Lan, Liu, Zhou & Zhou, 2024, and *T. wenshanensis* Jiang, Cao, Song, Yi & Yang, 2025);
3. Tip of pelvic fin reaching anus (vs not reaching anus in *T. tianlinensis*, *T. aluensis* Li & Zhu, 2000, and *T. tianeensis*);
4. Lateral line complete (vs incomplete in *T. langpingensis* Yang, 2013);
5. Posterior chamber of air bladder developed (vs reduced in *T. yangi* Jiang, Cao, Song, Yi & Yang, 2025);
6. Dorsal fin iii, 7 (vs iv, 9 in *T. cehengensis* Luo, Mao, Zhao, Xiao & Zhou, 2023, and iii, 9 in *T. rosa*).

##### Description.

Detailed morphometric data of *Triplophysa baishuijiangensis* sp. nov. specimens are summarized in Table 2 and Suppl. material 4. Dorsal fin iii, 7; pectoral fin i, 9–10; pelvic fin i, 6; anal fin ii, 5; 15–16 branched rays in the caudal fin; vertebrae: 4+34.

**Table 2.** Morphometric characters data of *Triplophysa baishuijiangensis* sp. nov.

Characters	Holotype	Holotype + Paratypes (2025000165-175)			
	KIZ 2025000165	Min	Max	Mean	SD
Standard length(SL)/mm	61,5	31,7	64,7	49,0	
Head length(HL)/mm	15,2	7,9	16,1	12,5	
**Percent of SL (%)**					
Body depth	14,5	10,9	15,9	13,5	1,5
Body width	11,7	7,9	12,6	10,1	1,7
Length of caudal peduncle	17,4	15,1	18,7	16,8	1,3
Caudal peduncle depth	7,6	5,6	8,3	7,1	0,8
Predorsal length	55,7	52,6	57,2	55,2	1,3
Prepectoral length	25,0	25,0	28,5	26,8	1,3
Prepelvic length	56,4	54,6	59,3	56,8	1,3
Preanal length	76,7	73,3	79,1	76,9	1,8
Dorsal fin base length	12,3	10,5	14,0	12,4	0,9
Dorsal fin length	21,4	19,4	22,8	20,8	1,2
Pectoral fin base length	5,9	3,8	5,9	4,6	0,7
Pectoral fin length	22,1	20,2	23,8	21,8	1,0
Pelvic fin base length	4,8	3,0	4,8	3,6	0,5
Pelvic fin length	16,0	14,1	17,0	15,6	1,0
Anal fin base length	7,3	7,3	9,8	8,5	0,7
Anal fin length	16,8	14,0	19,0	16,8	1,3
Caudal fin Length	21,9	20,7	26,2	22,6	1,6
Δ (upper - lower caudal fin lobe length)	2,7	1,0	3,2	2,2	0,7
pectoral–pelvic distance	19,2	23,4	33,3	29,9	3,7
pelvic–anal distance	13,5	13,6	20,0	17,1	2,1
vent–anal fin origin distance	2,6	2,6	6,7	4,6	1,3
HL	24,7	24,7	27,0	25,5	0,8
**Percent of HL (%)**					
Head depth at nape	50,1	41,0	50,3	45,7	3,3
Snout length	49,7	43,2	52,9	47,8	2,8
Eye diameter	6,1	2,9	6,1	4,8	1,2
Interorbital width	35,3	25,2	38,6	30,3	4,6
Preanterior nostril length	29,3	20,8	29,7	24,6	3,3
Distance between posterior nares	32,1	21,9	32,1	27,5	3,6
Upper jaw length	22,2	18,0	25,1	22,0	2,2
Lower jaw length	20,2	15,4	20,2	17,8	1,8
Mouth slit wide	26,6	23,5	30,5	26,5	2,3
Outer rostral barbel length	36,4	27,6	42,7	35,0	4,9
Internal rostral barbel length	23,3	19,2	25,0	22,1	2,1
Maxillary barbel length	34,9	25,3	35,0	30,7	3,3

Body elongated, anteriorly cylindrical, posteriorly compressed laterally behind dorsal-fin origin. Maximum body depth at tip of adpressed pectoral fins (corresponding to the swollen position of posterior swim bladder chamber). Snout slightly pointed. Eyes vestigial, reduced to black pigment spots.

Three pairs of barbels: outer rostral barbel longest, extending beyond posterior nostril; maxillary barbel shorter, extending past the eye spot; inner rostral barbel shortest, extending to the corner of mouth. Mouth inferior, arched, lower lip folded with medial V-shaped notch. Anterior and posterior nostrils closely connected, anterior nostril enclosed within a nasal valve that extends into a barbel-like tip, posterior nostril larger and without nasal valve.

Posterior edge of dorsal fin straight, distance from dorsal fin base to snout tip greater than to caudal fin base, dorsal fin starts before pelvic fins. Pectoral fins developed, arc-shaped, tips reaching or exceeding midpoint between dorsal and pectoral fin bases. Pelvic fins arc-shaped, slightly behind dorsal fin, extending to anus. Posterior edge of anal fin straight, separated from anus by a short distance. Caudal fin shallowly concave, upper lobe slightly longer than lower. Faint adipose crests on both dorsal and ventral sides of caudal peduncle, dorsal crest larger than ventral.

Two chambers of swim bladder, anterior chambers dumbbell-shaped and encased in bony capsule, posterior chamber developed, oval, and free within the abdominal cavity (Fig. 2G, H). Gill rakers not developed, nine gill rakers on first gill arch. Intestine short, length 52.0% of SL, bends downward behind the stomach and gradually narrows, stomach is U-shaped and enlarged.

##### Coloration.

Entire body lacks pigment and scaleless, lateral line complete. Live specimens semi-transparent, flesh-colored, internal organs vaguely visible ventrally. Fin rays white, fin membranes transparent (Fig. 4B). After fixation, body turns white, abdomen pale yellow. Eye spot color intensity varies, with some nearly invisible.

##### Sexual dimorphism.

All examined specimens lack thickened pads and furcella on the head and pectoral fins. However, males and females show significant differences in head shape. Male heads narrow sharply at the eye position, then taper gradually, resembling a “bell shape” with a blunt snout. Female heads taper gradually, resembling a “triangle” with a pointed snout (Fig. 3A, B). Consistent with the sexual dimorphism characteristics of *T. daqiaoensis*, described by Hou et al. (2010).

##### Distribution and habitat.

So far, the new species has only been found at the outlet of an underground river in Xiaoganxi Village, Yiliang County, Zhaotong City, Yunnan Province. Water of outlet is clear year-round (Fig. 4A). The new species is active and feeds near the outlet, rarely far from the outlet, responding to light but is insensitive to vibrations, and moves slowly and is easy to capture.

##### Etymology.

The specific epithet *baishuijiangensis* is in reference to the type locality of the new species: the core area of Baishuijiang National Aquatic Germplasm Resources Reserve for Endemic Fish. We propose the common English name “Baishuijiang high-plateau loach” and the Chinese name “Bái Shuǐ Jiāng Gāo Yuán Qiū (白水江高原鳅)”.

## ﻿Discussion

Currently documented cave-dwelling species of *Triplophysa* are predominantly distributed in karst landscapes of eastern Yunnan, northern Guangxi, southwestern Guizhou, and the Hunan-Chongqing border region. Hydrologically, with the exception of *T. wenshanensis*, *T. xichouensis*, and *T. gejiuensis* within the Red River system, all documented species inhabit tributaries of the Yangtze River (including Wujiang, Yuanjiang, and Hengjiang on the right bank) and Pearl River systems (predominantly Beipanjiang, Nanpanjiang, Hongshuihe, and Liujiang). The Yangtze River system harbors seven species compared to 33 species in the Pearl River system (Lan et al. 2004; Cao et al. 2025; Suppl. material 1). This biogeographical pattern reveals marked spatial heterogeneity: northern populations (Yangtze system) demonstrate lower species richness with scattered distributions, whereas southern populations (Pearl River system) exhibit higher species diversity and aggregation density (Fig. 5). This pattern likely reflects differential development of karst geomorphology between regions or uneven historical dispersal processes.

High congruence exists between the phylogenetic structure and geographic distribution pattern in hypogean species of *Triplophysa*. Phylogenetic analyses delineate two major clades within the hypogean group. Species of Clade I are distributed across eastern Yunnan, western Guizhou, and northwestern Guangxi regions, while species of Clade II occupy eastern territories relative to Clade I. The two distribution ranges are allopatric, with the extension line of lower reaches of the Beipanjiang River approximately serving as the east-west demarcation boundary between them (Figs 1, 5).

The trogloxene group (*Claea* spp.), sister to these hypogean group (Fig. 1), is currently found only in upper Yangtze reaches (Zhang et al. 2024; Fricke et al. 2025). As a lineage embedded within *Triplophysa*, *Claea* retains generic validity due to absent secondary sexual traits and processus dentiformis on the upper jaw, current evidence thus suggests the polyphyly of *Triplophysa* (Zhang et al. 2024; Lei et al. 2025). With more than 160 documented species, the taxonomic revision of *Triplophysa* is a significant challenge. Consequently, both the taxonomic status of *Claea* and whether the hypogean group of *Triplophysa* represents a distinct genus require further systematic study. Among the 43 cave-dwelling *Triplophysa* species, 16 species inhabit cave entrances, subterranean river outlets, or surface water bodies connected to subterranean systems (Suppl. material 1). Moreover, 33 species (77%) retain normal or partially degenerated eyes (Luo et al. 2023a; Lan et al. 2004; Cao et al. 2025; Suppl. material 2). Notably, *T. baishuijiangensis*, despite exhibiting extreme ocular degeneration and pigment loss, retains the ability to survive briefly outside the cave near its outlets, suggesting that some cave-dwelling *Triplophysa* species retain limited adaptability to surface environments. These species may periodically leave caves independently or be displaced by flooding events, resulting in short-distance dispersal to other suitable subterranean habitats. Interspecific genetic distances among cave-dwelling *Triplophysa* are relatively low (Suppl. material 3), with 13 species pairs (15 species in total) exhibiting genetic distances below 2.00%: *T. qingzhenensis* and *T. wudangensis* (1.62%), *T. macrocephala* and *T. longipectoralis* (1.51%), *T. wudangensis* and *T. rosa* (1.44%), *T. qingzhenensis* and *T. rosa* (1.42%), *T. longipectoralis* and *T. nasobarbatula* (1.24%), *T. rongduensis* and *T. zhenfengensis* (0.97%), *T. nasobarbatula* and *T. macrocephala* (0.80%), *T. longliensis* and *T. sanduensis* (0.71%), *T. tianeensis* and *T. fengshanensis* (0.35%), *T. guizhouensis* and *T. yaluwang* (1.45%), *T. ziyunensis* and *T. qingzhenensis* (1.73%), *T. ziyunensis* and *T. wudangensis* (1.45%), *T. ziyunensis* and *T. rosa* (1.54%). The relatively low genetic distances and geographic distance (Fig. 5) of these species pairs may suggest relatively recent isolation, speciation or introgression events (Liu et al. 2023; Yuan et al. 2023). Collectively, these findings suggest that cave-dwelling *Triplophysa* may have undergone rapid adaptive radiation and dispersal within the southwestern karst region. Furthermore, the growth rate of lineage numbers in cave-dwelling *Triplophysa* reached its peak during the late stages of differentiation (Yan 2018), which further corroborates the aforementioned viewpoint.

*Triplophysa baishuijiangensis* sp. nov. marks the first recorded cave loach in the Yangtze River basin within Yunnan and simultaneously the first documented typical cavefish in the Jinsha River system. Its discovery validates the presence of environmental conditions conducive to cavefish evolution in this basin. Field investigations conducted by authors reveal extensive karst development in Hengjiang and Chishui River basins, with the Chishui River basin exhibiting particularly dense subterranean cave systems and underground river networks, highlighting the potential cave biodiversity in these areas and warranting further investigation and research.

### ﻿Comparative material examined

***T. shilinensis***: KIZ1991000936–937, 59.1–59.3 mm SL, China: Yunan Province: Shilin County: Weiboyi Village.

***T. anshuiensis***: KIZ2012005746–5747, 64.7–68.4 mm SL, China: Guangxi Zhuang Autonomous Region: Lingyun County: Anshui Village.

***T. gejiuensis***: KIZ1978000982–985, 41.6–45.5 mm SL, China: Yunan Province: Gejiu City: Kafan Town.

***T. huapingensis***: KIZ2008007606–7608, 57.6–61.7 mm SL, China: Guangxi Zhuang Autonomous Region: Leye County: Huaping Town.

***T. nandanensis***: KIZ9110001135–1137, 53.2–79.0 mm SL, China: Guangxi Zhuang Autonomous Region: Nandan County: Liuzhai Town: Mayang Village: Xijiang River.

***T. longliensis***: KIZ2010002987–2988, KIZ2010003221, 75.9–97.9 mm SL, China: Guizhou Province: Longli County: Baisheng Village: Hongshuihe River.

***T. guizhouensis***: KIZ2017000346, KIZ2017000348, 73.5–110.3 mm SL, China: Guizhou Province: Huishui County: Baijin Village: Hongshuihe River.

***T. rosa***: KIZ2002005675, 53.8 mm SL, China: Chongqing City: Wulong County: Jiangkou Town: Tianxing Village: Wujiang River.

***T. tianlinensis***: KIZ2012005690–5691, KIZ2012005693, 48.7–84.7 mm SL, China: Guangxi Zhuang Autonomous Region: Tianlin County: Langping Town: Hongxing Village: Hongshui River.

***T. tianeensis***: KIZ2003005724–5729, 57.4–58.9 mm SL, China: Guangxi Zhuang Autonomous Region: Tiane County: Bala Town: Hongshui River.

***T. macrocephala***: KIZ2010003078, KIZ2010003080–3081, 50.4–54.7 mm SL, China: Guangxi Zhuang Autonomous Region: Nandan County: Lihu Town: Renguang Village: Xijiang River.

***T. luochengensis***: KIZ2014005967–5968, 45.0–53.8 mm SL, China: Guangxi Zhuang Autonomous Region: Luocheng County: Jicheng Village: Hongshuihe River.

***T. longipectoralis***: KIZ2001004573–4574, KIZ2001004579, 46.9–51.9 mm SL, China: Guangxi Zhuang Autonomous Region: Hechi City: Huanjiang County: Xunle Town: Liujiang River.

***T. xichouensis***: KIZ2013600381–382, 57.4–61.4 mm SL, China: Yunan Province: Xichou County: Xisa Town: Red River.

## Supplementary Material

XML Treatment for Triplophysa baishuijiangensis

## ﻿Additional information

### Use of AI

No use of AI was reported.

### Data availability

All of the data that support the findings of this study are available in the main text or Supplementary Information.

## Supplementary materials

Supplementary material 1 Species distribution table Data type xlsx https://binary.pensoft.net/file/1422148This dataset is made available under the Open Database License (http://opendatacommons.org/licenses/odbl/1.0/). The Open Database License (ODbL) is a license agreement intended to allow users to freely share, modify, and use this Dataset while maintaining this same freedom for others, provided that the original source and author(s) are credited.Min Shi

Supplementary material 2 Morphological comparison table of cave-dwelling *Triplophysa* Data type xlsx https://binary.pensoft.net/file/1422149This dataset is made available under the Open Database License (http://opendatacommons.org/licenses/odbl/1.0/). The Open Database License (ODbL) is a license agreement intended to allow users to freely share, modify, and use this Dataset while maintaining this same freedom for others, provided that the original source and author(s) are credited.Min Shi

Supplementary material 3 Cyt b Genetic K2p distance of 43 sequences Data type xls https://binary.pensoft.net/file/1422151This dataset is made available under the Open Database License (http://opendatacommons.org/licenses/odbl/1.0/). The Open Database License (ODbL) is a license agreement intended to allow users to freely share, modify, and use this Dataset while maintaining this same freedom for others, provided that the original source and author(s) are credited.Min Shi

Supplementary material 4 Supplementary material 4-original measurement data of *Triplophysa baishuijiangensis* sp. nov. Data type xlsx https://binary.pensoft.net/file/1422150This dataset is made available under the Open Database License (http://opendatacommons.org/licenses/odbl/1.0/). The Open Database License (ODbL) is a license agreement intended to allow users to freely share, modify, and use this Dataset while maintaining this same freedom for others, provided that the original source and author(s) are credited.Min Shi
